# A Systematic Review of the Psychometric Properties of the Geriatric Anxiety Inventory

**DOI:** 10.1093/geroni/igaa057.1185

**Published:** 2020-12-16

**Authors:** Philippe Landreville, Alexandra Champagne, Patrick Gosselin

**Affiliations:** 1 Université Laval, Québec, Quebec, Canada; 2 Université de Sherbrooke, Sherbrooke, Quebec, Canada

## Abstract

The Geriatric Anxiety Inventory (GAI) is a widely used self-report measure of anxiety symptoms in older adults. Although much research has been conducted on the psychometric properties of the GAI, previous reviews have examined only a small proportion of studies and have not evaluated the methodological quality of this work. In view of this, we conducted a systematic review of the psychometric properties of the GAI and it’s short form (GAI-SF). Relevant studies (N = 31) were retrieved through a search of electronic databases (Pubmed, PsycINFO, CINAHL, EMBASE and Google Scholar) and a hand search. The methodological quality of the included studies was assessed by two independent reviewers using the ‘‘COnsensus-based Standards for the selection of health status Measurement INstruments’’ (COSMIN) checklist. Based on the COSMIN checklist, internal consistency and test reliability were mostly rated as poorly assessed (63% and 72.7% of studies, respectively) and quality of studies examining structural validity was mostly fair (60% of studies). Both the GAI and GAI-SF showed adequate internal consistency and test-retest reliability. Convergent validity indices were highest with measures of generalized anxiety and lowest with instruments that include somatic symptoms. Substantial overlap with measures of depression was reported. While there is no consensus on the factorial structure of the GAI, the short version was found to be unidimensional. Our review therefore suggests that the GAI and GAI-SF have satisfactory psychometric properties while indicating that future efforts should aim to achieve a higher degree of methodological quality.

